# Supplementary material for: Lipid droplet targeting of the lipase coactivator ABHD5 and the fatty liver disease-causing variant PNPLA3 I148M is required to promote liver steatosis
Source: J Biol Chem. 2025 Jan 13;301(2):108186. doi: 10.1016/j.jbc.2025.108186 (PMC11849118; doi:10.1016/j.jbc.2025.108186)
Supplement: Supporting information [file mmc1.docx]

**Lipid droplet targeting of the lipase co-activator ABHD5 and the fatty liver disease-causing variant PNPLA3 I148M is required to promote liver steatosis**

Grace Teskey^1^, Nivedita Tiwari^1^, Andrew J. Butcko^1,2^, Amit Kumar^3^, Anuradha Yadav^3^, Yu-ming M. Huang^3^, Christopher V. Kelly^3^, James G. Granneman^4^ James W. Perfield^5^ and Emilio P. Mottillo^1,2,^*

**SUPPORTING INFORMATION**

**Figure S1**

**Figure S2**

**Figure S3**

**Figure S4**

**Figure S5**

**Table S1**

**Movie 1 legend**

**Movie 2 legend**

**
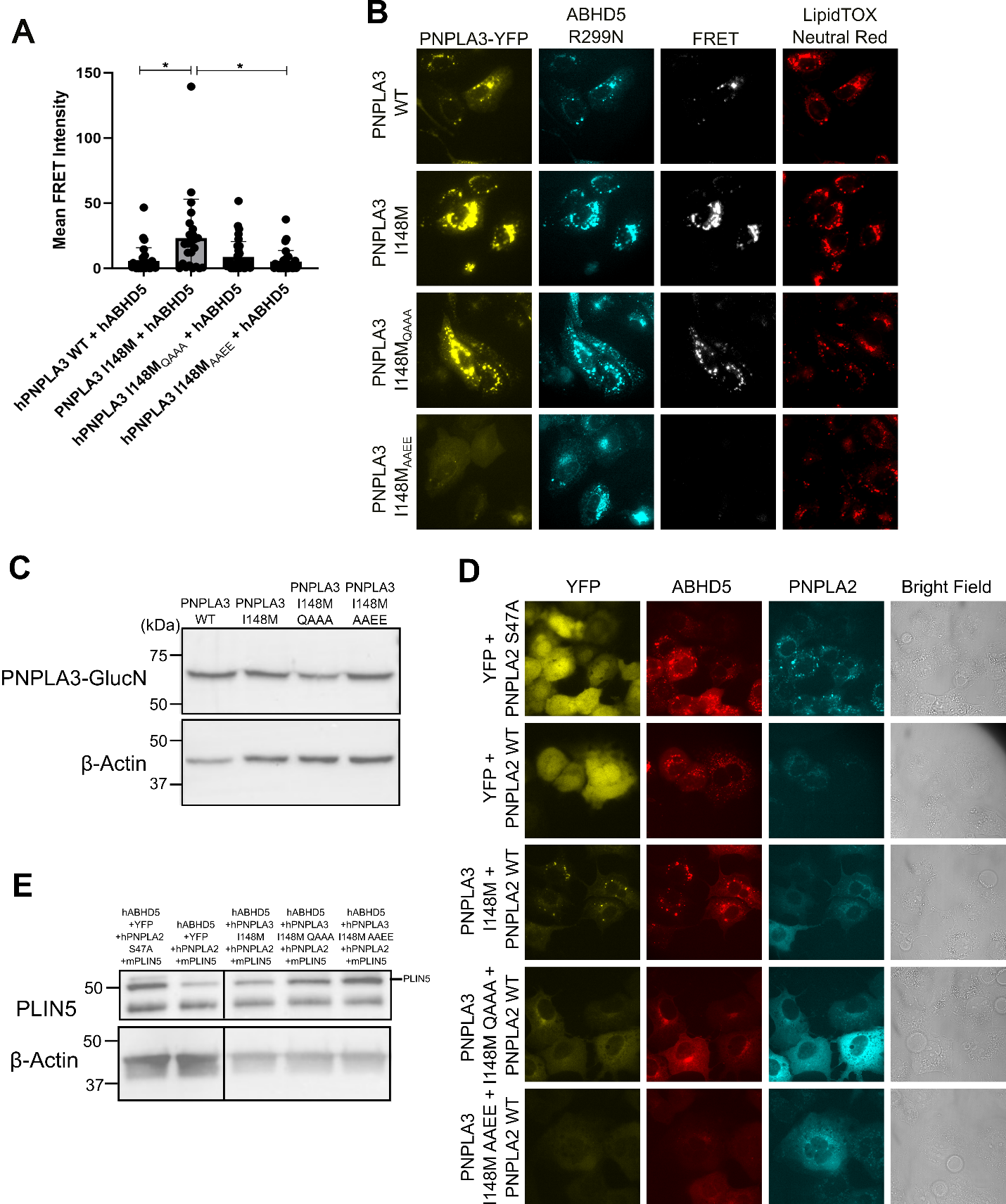
**

**Figure S1:** (**A**) Quantification of the mean nFRET intensity of individual cells from Figure 1C. *p <0.05 as determined by Kruskal-Wallis test with Dunn’s multiple comparison. (**B**) Fluorescent imaging and FRET analysis of U2OS cells transfected with EYFP-tagged WT hPNPLA3 or I148M and ECFP-tagged WT mABHD5 or mABHD5-R299N. Cells were treated with 0.2mM oleic acid overnight and stained with LipidTOX Neutral Red. Images are representative of three consecutive experiments with three technical replicates per trial. Scale bar, 10 µm. (**C**) Western blot of PNPLA3-GlucN and β-Actin expression in HEK293A cells transfected with plasmids used for protein complementation assay. Blot shows similar PNPLA3-GlucN expression for variants and is representative of four separate western blot experiments. (**D**) Representative panel of images used for blinded quantification of transfected cells after fluorescent imaging of COS-7 cells transfected with EYFP-tagged PNPLA3 I148M or mutants, mCherry-tagged ABHD5, ECFP-tagged PNPLA2 WT or ATGL_S47A_, and PLIN5 with visible lipid droplets in brightfield. Cells were treated with 0.2mM oleic acid overnight. Images are representative of three trials with three technical replicates per trial. Scale bar, 10 µm. (**E**) Western blot of PLIN5 and β-Actin protein expression from cells transfected similarly to experiment 1F.


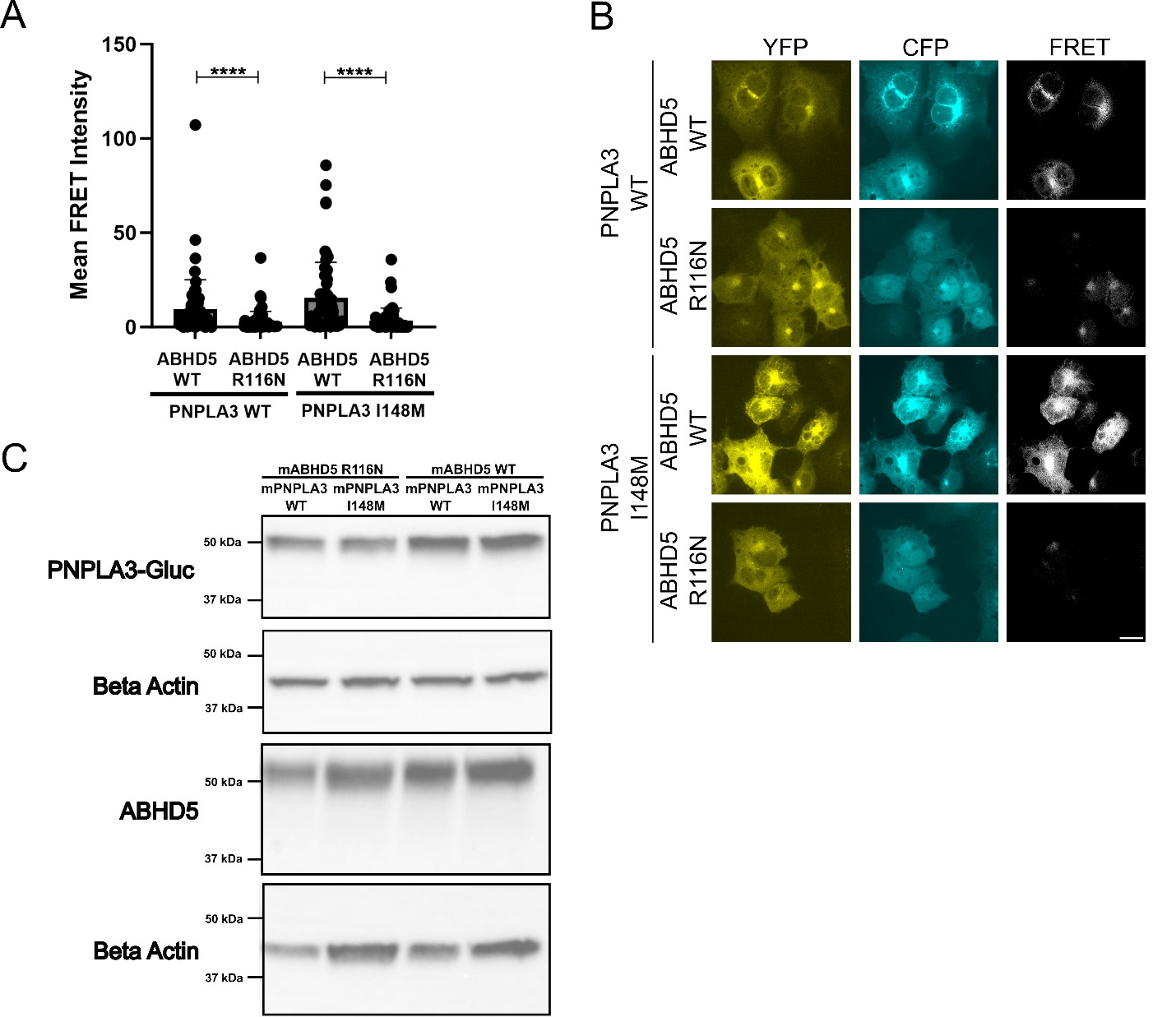


**Figure S2:** (**A**) Quantification of the mean nFRET intensity of individual cells for WT PNPLA3 and PNPLA3 I148M with WT ABHD5 or R116N from Figure 2A. ****p <0.0001 as determined by Kruskal-Wallis test with Dunn’s multiple comparison. (**B**) Fluorescent imaging and FRET analysis of COS-7 cells transfected with YFP-tagged mPNPLA3 (WT or I148M variant) and ECFP-tagged mABHD5 (WT or R116N variant). Experiment was performed similar to Figure 2A except the cells were not treated with oleic acid overnight demonstrating the localization of ABHD5 R116N is primarily cytosolic. Images are representative of results seen in three consecutive experiments. (**C**) Western blot of PNPLA3-GlucN and β-Actin and ABHD5-GlucC and β-Actin expression in HEK293A cells transfected with plasmids used for protein complementation assay. Blot shows that PNPLA3-GlucN and ABHD5-GlucC expression is present and similar across experimental treatments.


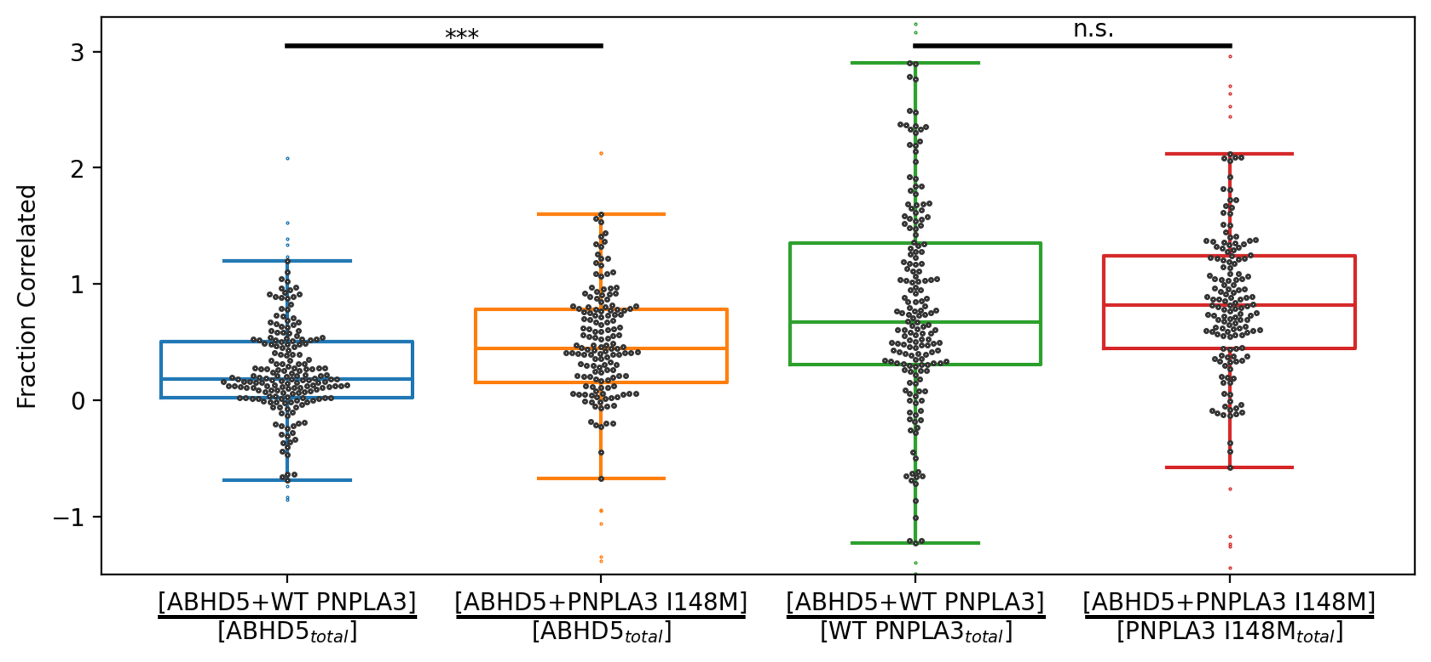


**Figure S3:** Analysis of the correlation amplitudes revealed the fraction of each protein that is part of a multi-color protein cluster through calculating G_0_^Cross^ / ­G­_0_^Auto^. ABHD5 is more likely to co-diffuse with PNPLA3 I148M than with WT PNPLA3 (p = 0.0005). The WT and the variant PNPLA3 are indistinguishably likely to be co-diffusing with ABHD5. Noise within each collected scan and uncertainties of the fits resulted in a wide distribution of individual scan fit results (symbols). Boxplots show the median and extent of the data (lines).


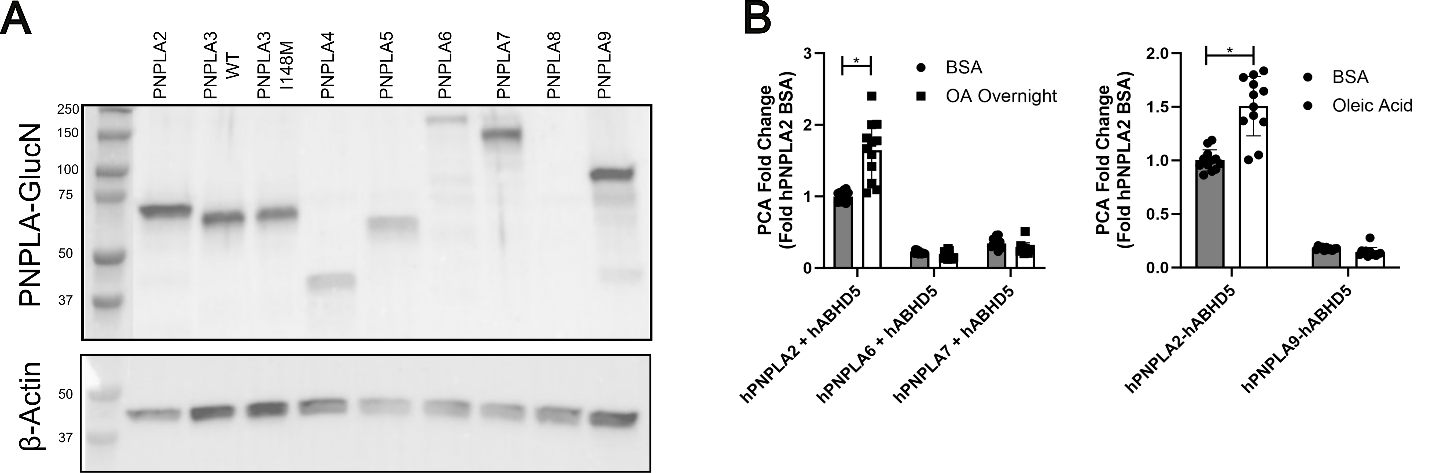


**Figure S4:** (**A**)Western blot showing expression of PNPLA proteins with GlucN tag and β-Actin. With the exception of PNPLA8, all PNPLAs were expressed in the protein complementation assays. (**B**) Gluc PC assay of HEK293A cells showing the lack of interaction of GlucN-tagged PNPLA6, PNPLA7 and PNPLA9 with GlucC-tagged ABHD5, both at baseline and after overnight 0.2mM oleic acid treatment. Data are from 3 trials with 4 technical replicates per trial. Statistics calculated using Two-way ANOVA with Sidak’s multiple comparison test (*p<0.05).


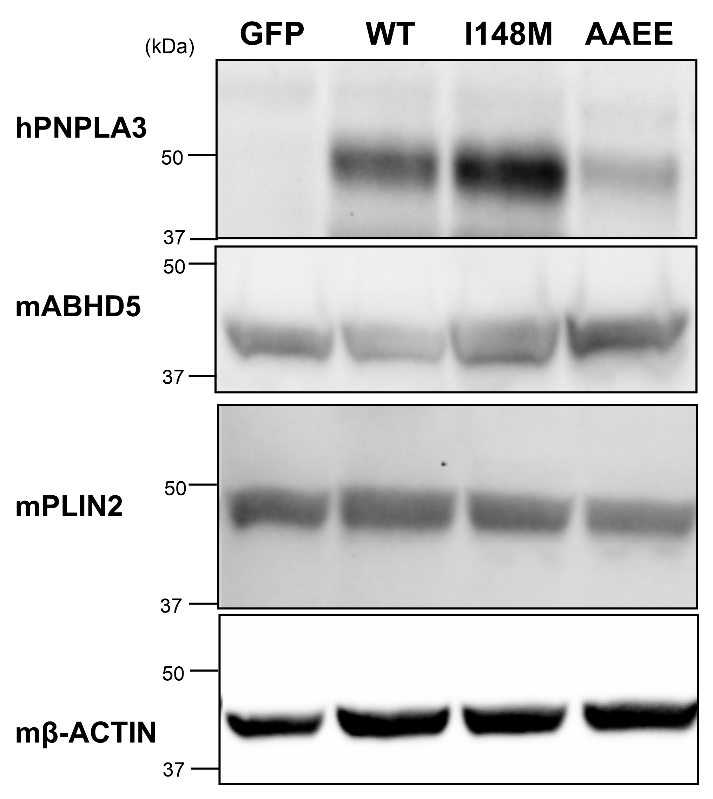


**Figure S5.** Western blot of total protein levels from Figure 6C for hPNPLA3, mABHD5, mPLIN2 and mβ-Actin.

**Movie 1:** CGMD simulation illustrating PNPLA3 association with the LD. PNPLA3 is displayed in green, with three regions (aa 80-88, 147-155, and 200-225) that interact directly with the LD highlighted in red. The helix of aa 345-368 is shown in orange, while the C-terminus (aa 369-481) is shown in blue. The LD is represented with phospholipid heads in brown and tails in cyan, while TAG is depicted in gray.

**Movie 2:** Snapshots from GaMD simulations showing PNPLA3 in solution and the PNPLA3-LD complex. The GaMD snapshots of PNPLA3 in solution and PNPLA3 bound to the LD, both captured around 1000 ns, are aligned. The PNPLA3 protein is shown in pink for the solution state and lime green for the membrane complex. Regions of PNPLA3 that form direct interactions with the LD, including aa 80-88, 147-155, 200-225, and 345-481, are highlighted in red for the solution state and dark green for the membrane complex. The LD includes phospholipid heads in brown, tails in cyan, and TAG in gray.

| **Overlap Extension PCR Primers** | |
| --- | --- |
| Primer Name | Primer Sequence 5'-3' |
| hPNPLA3 I148M ^370^QAAA^373^ - Forward | AATCTGCCATTGCGATTGTCCAGGCAGCGGCGACATGGCTTCCAGATATGCCC |
| hPNPLA3 I148M ^370^QAAA^373^ - Reverse | GGGCATATCTGGAAGCCATGTCGCCGCTGCCTGGACAATCGCAATGGCAGATT |
| hPNPLA3 I148M ^370^AAEE^373^ - Forward | AATCTGCCATTGCGATTGTCGCAGCGGAGGAGACATGGCTTCCAGATATGCCC |
| hPNPLA3 I148M ^370^AAEE^373^ - Reverse | GGGCATATCTGGAAGCCATGTCTCCTCCGCTGCGACAATCGCAATGGCAGATT |
| **Cloning PNPLAs with GLucN** | |
| Primer Name | Primer Sequence 5'-3' |
| PNPLA6 - Forward | CGCGGCTAGCGCCACCATGGGGACATCGAGTCACGGGCT |
| PNPLA6 - Reverse | CGCGGGATCCGGGGCATCTGTGGCTGAGCCGGGC |
| PNPLA7 - Forward | CGCGGCTAGCGCCACCATGGAGGAAGAGAAAGATGACAGCC |
| PNPLA7 - Reverse | CGCGAAGCTTGGCCCGTCCTGGTCAGAGGAGCC |
| PNPLA9 - Forward | CGCGAAGCTTGCCACATGCAGTTCTTTGGCCGCCTGG |
| PNPLA9 - Reverse | CGCGACCGGTGGGGGTGAGAGCAGCAGCTGGATGAG |
| **hPNPLA3 C-terminus Amplification for PNPLA4 Fusion** | |
| Primer Name | Primer Sequence 5'-3' |
| PNPLA3 320 - Forward | CCAACCGGTCCCCAGGCTCGCTACAGCACT |
| PNPLA3 481 - Reverse | CCAACCGGTGGCAGACTCTTCTCTAGTGAAAAACTGGGAAA |

**Table S1: List of primers used.**
